# Hypertension Development by Midlife and the Roles of Premorbid Cognitive Function, Sex, and Their Interaction

**DOI:** 10.1161/HYPERTENSIONAHA.118.12164

**Published:** 2019-02-19

**Authors:** Drew M. Altschul, Christina Wraw, Geoff Der, Catharine R. Gale, Ian J. Deary

**Affiliations:** 1 From the Department of Psychology (D.M.A., C.W., I.J.D.), University of Edinburgh, United Kingdom; 2 Centre for Cognitive Ageing and Cognitive Epidemiology (D.M.A., C.W., C.R.G., I.J.D.), University of Edinburgh, United Kingdom; 3 MRC/CSO Social and Public Health Sciences Unit, University of Glasgow, United Kingdom (G.D.); 4 MRC Lifecourse Epidemiology Unit, University of Southampton, Southampton General Hospital, United Kingdom (C.R.G.).

**Keywords:** cognition, humans, hypertension, income, sex

## Abstract

Higher early-life cognitive function is associated with better later-life health outcomes, including hypertension. Associations between higher prior cognitive function and less hypertension persist even when accounting for socioeconomic status, but socioeconomic status-hypertension gradients are more pronounced in women. We predicted that differences in hypertension development between sexes might be associated with cognitive function and its interaction with sex, such that higher early-life cognitive function would be associated with lower hypertension risk more in women than in men. We used accelerated failure time modeling with the National Longitudinal Study of Youth 1979. Cognitive function was assessed in youth, when participants were aged between 14 and 21 years. Of 2572 men and 2679 women who completed all assessments, 977 men and 940 women reported hypertension diagnoses by 2015. Socioeconomic status in youth and adulthood were investigated as covariates, as were components of adult socioeconomic status: education, occupational status, and family income. An SD of higher cognitive function in youth was associated with reduced hypertension risk (acceleration factor: ĉ=0.97; 95% CI, 0.96–0.99; *P*=0.001). The overall effect was stronger in women (sex×cognitive function: ĉ=0.97; 95% CI, 0.94–0.99; *P*=0.010); especially, higher functioning women were less at risk than their male counterparts. This interaction was itself attenuated by a sex by family income interaction. People with better cognitive function in youth, especially women, are less likely to develop hypertension later in life. Income differences accounted for these associations. Possible causal explanations are discussed.

## Abstract 2

Supplemental Digital Content is available in the text.

Hypertension has been consistently linked to cardiovascular diseases, such as coronary artery disease (CAD) and stroke.^1^ It is also a risk factor for neurocognitive conditions, such as early cognitive decline, vascular dementia, and possibly Alzheimer disease.^2^ Accelerated cognitive decline is associated with lower well-being and higher morbidity and mortality, and as cognitive function worsens, the clinical conditions of mild cognitive impairment and dementia can develop.^3^ Some of hypertension’s negative impacts on cognitive function have likely causal pathways: hypertension disrupts cerebral blood vessel structure and function and is associated with stroke in relevant white matter regions.^4^ Worldwide, hypertension, age-related cognitive decline, and dementia are on the rise.^4,5^

Typically, hypertension is thought of as a risk factor for later cognitive decline. However, there is also evidence for the relationship operating in the opposite direction, that is, that higher cognitive function in youth is associated with having lower risk of developing hypertension^6^ and experiencing hypertension-related stroke and coronary artery events later in life.^7^ These findings are part of a field known as cognitive epidemiology, which has found that higher cognitive function in early life is associated with lower risk of a number of physical and mental ailments later in life.^8–12^

Men are more likely to develop cardiovascular conditions than women^13^—a reason why men have been the subject of more intervention studies than women.^14^ Nevertheless, cardiovascular disease is the leading cause of death in both women and men.^15^ Some differences in hypertension are biologically based on differences between men and women, for example, through hormones and gene dosage from the sex chromosomes, and these differences are consistent across different countries and ethnic groups.^14^ Additionally, traditional sex roles are associated with men behaving in ways (eg, higher smoking rates) that increase their risk for physical health conditions, including hypertension.^16^

Previous work on the cognitive epidemiology of CAD and stroke events found a significant interaction between sex and cognitive function in youth: individuals with higher cognitive function were at lower risk for CAD and stroke, and the associations were stronger in women.^7^ However, the numbers of events in studies of CAD have been small.^6,7^ Here, we hypothesized that the development of hypertension—a condition that becomes increasingly common with age and is related to cardiovascular health and cognitive impairment—could differ by sex, such that higher early-life cognitive function is associated with lower risk of hypertension in women than it is in men.^7^ We tested this hypothesis using the US National Longitudinal Survey of Youth 1979 (NLSY79), following prior work linking cognitive function in youth and physical health in midlife in this sample.^6^ Socioeconomic factors, in particular education, have been implicated as mediators in the relationship between cognitive function in youth and cardiovascular risk^17–19^; these were examined in the present study, both as potential mediators and moderators.

## Methods

### Materials and Data Availability

Anonymized data and materials have been made publicly available from the United States Bureau of Labor Statistics National Longitudinal Surveys website and can be accessed at www.nlsinfo.org/investigator. The R code used in the present study is available on request.

### Participants

The NLSY79 was initially sampled from noninstitutionalized people aged 14 to 21 years, living in the United States.^20^ The study consisted of 12 686 original participants and was representative of the population at the time; 16% of participants were Hispanic, 25% were black, and 59% were neither black nor Hispanic.

The initial interview took place in 1979, and respondents were reinterviewed annually until 1994, and surveys were conducted biennially after. For the health modules, in which the hypertension diagnosis data were collected, not all individuals were surveyed every 2 years. Rather, each individual participating in the module(s) was surveyed for that module during the wave(s) when they were closest to 40 and 50 years of age, for each respective health module. The most recent data come from the 2014 health survey.

### Hypertension Diagnosis

Respondents were asked whether they had ever been told by a doctor that they had high blood pressure or hypertension. If respondents answered yes, they were asked for the month and year that this was first diagnosed. Right censored survival data were thus constructed as starting at the date of cognitive function measurement and ending at the time of hypertension diagnosis or being censored at the most recent date of data collection in which they took part. Individuals who did not provide information on hypertension diagnosis were not included in the analyzes, nor were individuals with hypertension before the study inception, because these cases more likely represent a congenital condition.^21^ Kaplan-Meier survival curves were plotted to visualize the effect of different variables and interactions on hypertension diagnosis.

### Cognitive Function

General cognitive function was assessed in the NLSY79 via the Armed Forces Qualifications Test (AFQT), scored using the 1989 renorming.^22^ The test was given in 1980, when participants were between 15 and 22 years of age; these tests’ scores reflect premorbid cognitive function. The scores were derived from 4 subtests that assessed arithmetic reasoning, mathematical knowledge, word knowledge, and paragraph comprehension. The AFQT is a valid and reliable measure of cognitive function, having been associated with outcomes including academic achievement and job performance.^23,24^ To be consistent with previous work in this sample,^6,25^ we used the *Z* scored AFQT percentile score, taken from The Bell Curve website.^26^

### Covariates

Sex was originally determined by observation, and if it was not obvious, participants were asked directly by the interviewer during the initial survey in 1979. Every case was checked, and in 45 cases corrected, by the National Opinion Research Center in 1986. Men were coded as the reference level, that is, 0, and women were coded as 1.

Several variables were incorporated as controls into progressive models. The age when the first interview was conducted in 1979 was included to control for lower test performance in younger individuals. Socioeconomic status (SES) in youth, that is, parental SES, was included to control for confounding effects of an individual’s rearing circumstances. Individuals from higher SES background may have access to more resources and benefit from higher cognitive functions in this way, although the existing literature suggests that these effects are slight.^27^

Adult SES, on the contrary, can have a much larger impact on associations between early-life cognitive function and later-life health.^27^ We included adult SES as a variable of interest, although the mechanisms relating adult SES, cognitive function, and health are debated.^12^ Adult SES is often theorized to have a mediating effect between cognitive function and health, but adult SES is also inherited: there are genetic correlations between cognitive function and SES,^28^ and substantial environmental circumstances can carryover from one generation to the next.^29^ Including adult SES allows us to control for potential confounding, for example, from inherited privilege, and consider the portion of adult SES that may mediate the relationship between early-life cognitive function and hypertension diagnosis. Adult SES is composed of adult measures of family income, education, and occupational status, each of which could have a different confounding or mediating effect. Thus each was also analyzed independently from the composite adult SES variable.

Youth SES and adult SES were averages of z-transformed income, education, and occupation status variables.^26^ To calculate youth SES, participants’ parents’ information was used; to calculate adult SES, individuals’ information from surveys from 2012 to 2014 was used. A higher SES value indicates more socioeconomic advantage.

The adult income variable was the total net family income in the past year, which was also log- and z-transformed to be consistent with earlier work.^6,26^ Adult education was the highest grade completed by the most recent wave of the study. Occupation status was derived as a continuous variable using an updated version of the Duncan Socioeconomic Index.^30,31^

### Statistical Analyses

All analyses were conducted using accelerated failure time (AFT) regression models—a form of survival analysis that is fully parametric and not limited by the assumptions of proportional hazard modeling, which these data did not satisfy (Table S1 in the online-only Data Supplement).^32^ With selection of the best parametric distribution, AFT models also allow for better fit and more accurate inferences.^33^ Complete case and multiply-imputed analyses using the same predictor variables yielded the same findings in previous work.^6^ A similar pattern of missing values could be expected in the following analyses; therefore, only complete cases were analyzed in the present study.

The outcome of AFT models was the event of a hypertension diagnosis and, if such a diagnosis was given, the date of the diagnosis. The log-logistic distribution was used as the error distribution in all models because it consistently produced better fit than the alternatives (Weibull, Gompertz, log-normal, and exponential distributions). The first model was our base model and included cognitive function, sex, and age of testing in youth. The second model introduced an interaction between sex and cognitive function, that is, asking the question of whether there was a stronger association in men or women between cognitive function in youth and hypertension by middle age. Model 3 added SES in youth as a covariate, and model 4 added adult SES to model 3. Because adult SES is composed of distinct subcomponents, that is, income, education, and occupational status, it has been informative to analyze the effect of each variable independently,^6^ to investigate possible mechanisms. Models 5 through 7 broke down adult SES into its constituent parts, adding each in isolation to model 3 to examine the statistical effects of adult SES in greater detail. Model 8 investigates the specific importance of income and its interaction with sex.

For all models, acceleration factors (ĉ) were presented, with 95% CI, as the quantification of the regression coefficients that result from AFT modeling. A variable’s acceleration factor represents the degree to which an event, that is, hypertension diagnosis, occurs sooner than it would on average, which is the reference level for categorical variables (eg, male, for sex) or the mean for continuous variables. If ĉ >1, the acceleration is more than average, meaning that the positive value of this variable increases the probability that the individual will be diagnosed with hypertension. If ĉ <1, the opposite is true, and a positive value of the variable will decrease hypertension risk, relative to the average. Results were expressed per SD of the exposure, that is, the AFQT score.

## Results

**Figure 1.**
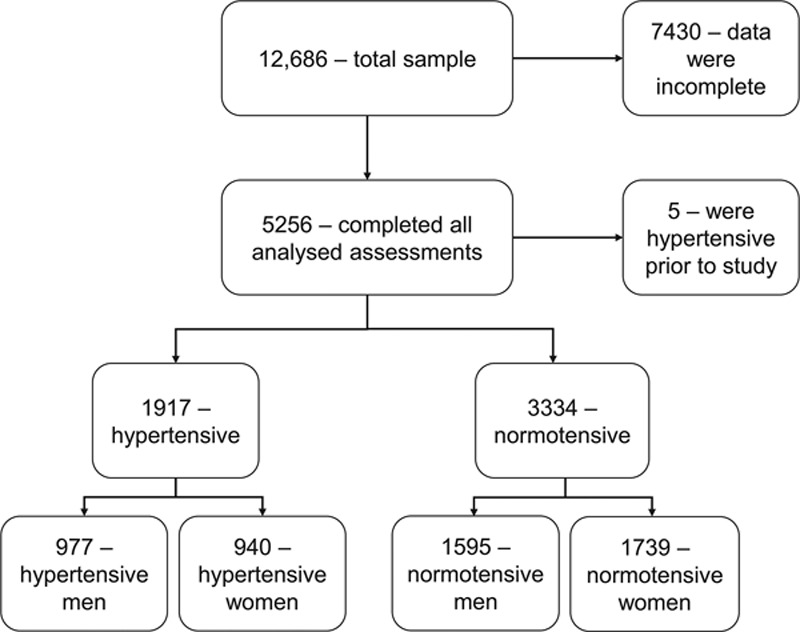
Flowchart of National Longitudinal Survey of Youth (NLSY) participants analyzed in this study. From the full NLSY sample, individuals were only analyzed if they had complete sex, cognitive function, youth socioeconomic status (SES), and adult SES data and hypertension diagnosis information from either the age 40 or 50 health module.

A flowchart of individual participation and hypertension status is presented in Figure 1. Of the original sample of 12 686 individuals, data were incomplete for 7430, which mostly consisted of individuals who did not participate in health modules for either age 40 or 50 years. Five more were hypertensive before the NLSY79 began. This yielded an analytic sample of 5251; 1917 of these individuals were diagnosed with hypertension.

Descriptive data for analyzed variables are presented in Table 1. Expanded sample characteristics can be found in the study by Wraw et al^6^ (Table 1). In ecologically relevant terms, adult annual incomes in the analytic sample ranged from $1811 to $595 986, with a mean of $82 989; years of education ranged from only having completed the third grade to >8 years of college, with a mean of 13.5 years of education stating from the first grade. Overall, the individuals in our analytic sample experienced slightly better socioeconomic circumstances in youth and adulthood than did the individuals who were missing data and not included in our analyses (Table S2); the variable means in each subsample were between 0.05 and 0.59 of an SD from the other. Contrary to some expectations,^34,35^ prevalence and average age of diagnosis of hypertension were highly comparable across men and women, in both the full and analytic sample. The higher proportion of hypertension diagnoses in the analytic sample reflects the older age of this subsample.

**Table 1.**
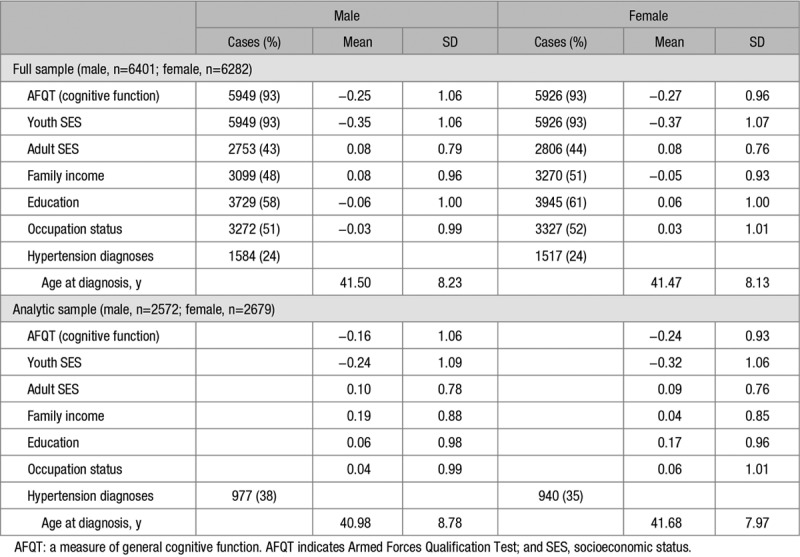
Descriptive Statistics of Explanatory, Control, and Outcome Variables, Split by Sex

Using cognitive function as a continuous variable, in our first model (Table 2), we found main effects of cognitive function (ĉ=0.96; 95% CI, 0.95–0.97; *P*<0.001), indicating that higher functioning individuals were less likely to develop hypertension; sex (ĉ=0.97; 95% CI, 0.95–0.99; *P*=0.019), indicating that women were less likely to become hypertensive; and survey age in youth (ĉ=0.99; 95% CI, 0.98–1.00; *P*=0.002), which could be because of older individuals scoring higher on the AFQT. In subsequent analyses, we added a sex by cognitive function interaction to our AFT models (Table 2). We found an interaction between sex and cognitive function (ĉ=0.97; 95% CI, 0.96–0.99; *P*=0.001), indicating that the cognitive function and hypertension association were stronger in women than men.

**Table 2.**
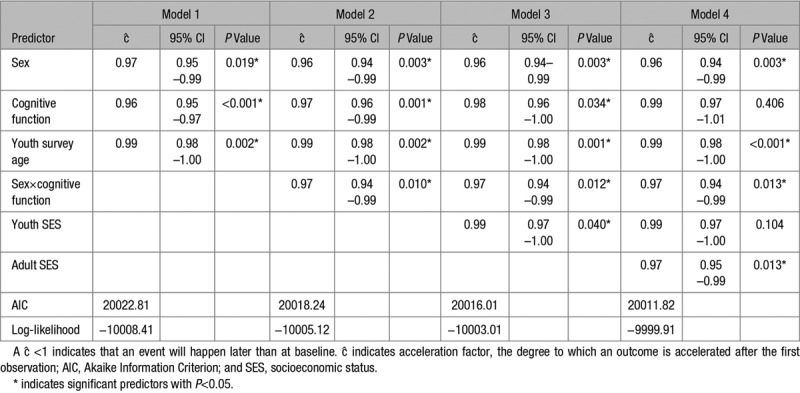
Accelerated Failure Time Models of Hypertension, Predicted by Sex, Cognitive Function, and SES Variables

**Figure 2.**
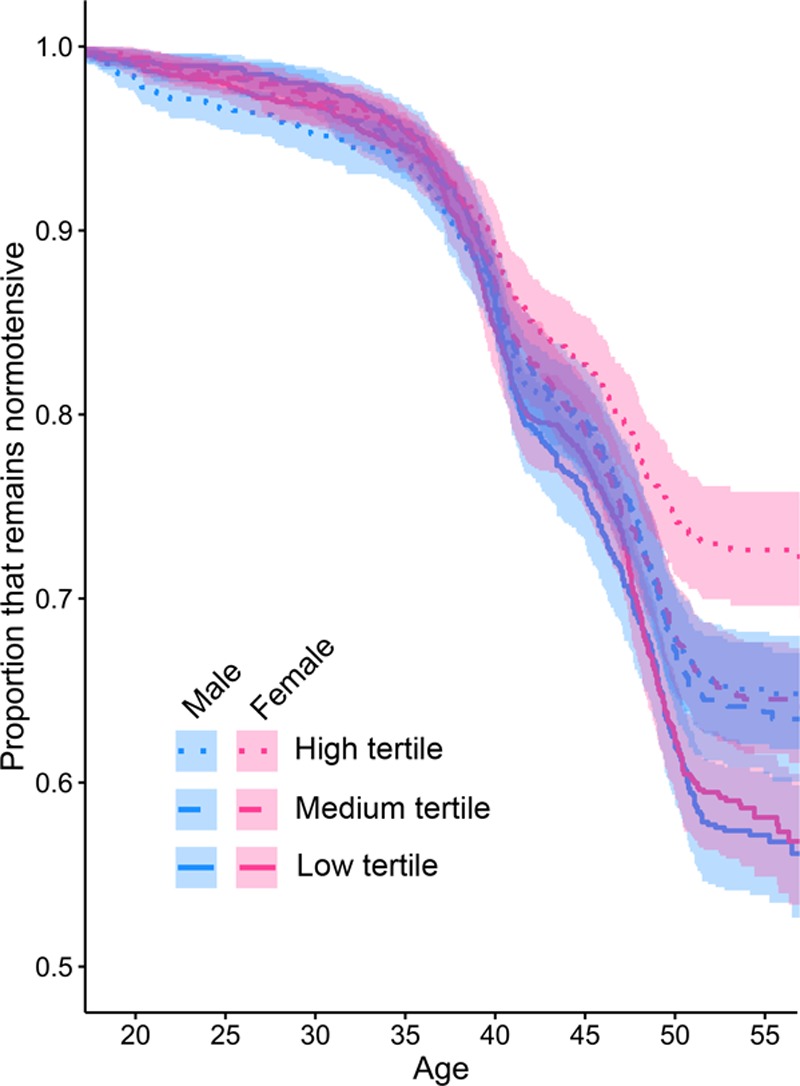
Kaplan-Meier curves of time to hypertension diagnosis. For visualization purposes, cognitive function across all individuals was divided into tertiles. Individuals in these tertiles were subdivided by sex, producing 6 curves. The band around each curve is the 95% confidence region.

Kaplan-Meier curves (Figure 2) illustrate the interaction between cognitive function and sex and the relationship with hypertension diagnosis (Figure 2). We note that, although cognitive function is divided into tertiles in Figure 2 for the purpose of illustration, the analyses were conducted with cognitive function as a continuous variable. In women, there are 3 distinct curves for hypertension risk (Figure 2); by their 50s, those women with high cognitive function in youth had a lower risk of hypertension than average (mid) cognitive scorers, who are in turn at lower risk than those with low cognitive function from youth. In men, the high and average cognitive scorers from youth have similar risk of hypertension by middle age, and both have lower risk than lower cognitive scorers. In addition to these within-sex observations, Figure 2 shows between-sex differences, that is, higher functioning women were less likely to be diagnosed with hypertension than higher functioning men.

Adding SES from youth had no effect on this interaction; it was not itself a predictor of hypertension diagnosis nor did it interact with sex (Table S3). Adding adult SES attenuated the main effect of cognitive function (ĉ=0.99; 95% CI, 0.97–1.01; *P*=0.406) but did not affect the interaction with sex. Adult SES also predicted hypertension development (ĉ=0.97; 95% CI, 0.95–0.99; *P*=0.013); higher SES individuals were less likely to be diagnosed with hypertension. We also fit the equivalent model separately in men and women (Table S4). These models confirmed the effects of our sex×cognitive function models, as significant effects of cognitive function and adult SES were present in women but not men.

Of the adult SES subcomponents, only income was significant (Table 3); in this model, the sex by cognitive function interaction remained significant. Moreover, education and occupation status did not seem to individually predict hypertension diagnoses, independently or as a part of the adult SES composite. The Akaike Information Criterion—a measure of model fit^36^—for model 5 in Table 2 indicated that the adult family income model was a better fit than the model that used composite SES, as well as the models using occupation and education.

**Table 3.**
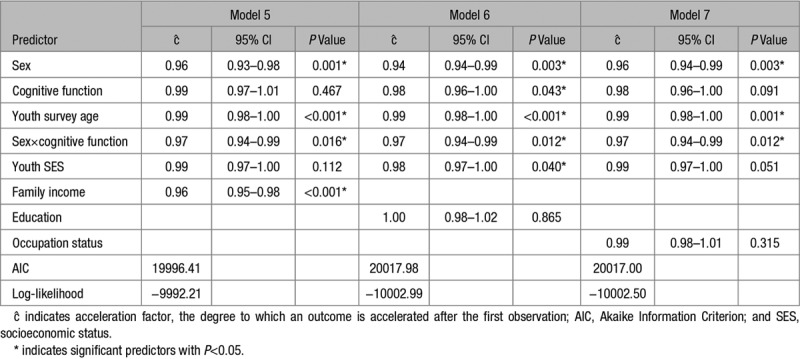
Accelerated Failure Time Models of Hypertension, Adding Individual Adult SES Predictors

To test whether income differences between sexes could explain the sex by cognitive function interaction, we added a sex by family income interaction to model 5. The model (Table 4) indicated that women with higher family income are less likely to develop hypertension, and the inclusion of this interaction reduced the acceleration factor of the sex by cognitive function interaction from 0.97 (95% CI, 0.94–0.99) to 0.98 (95% CI, 0.95–1.01).

**Table 4.**
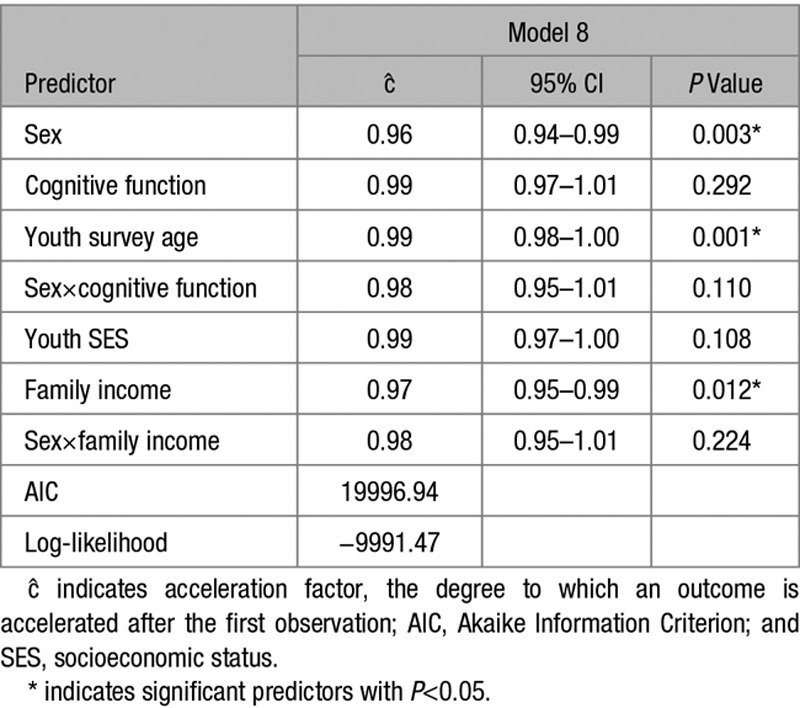
Accelerated Failure Time Model of Hypertension, Adding Sex by Income Interactions

Whereas the sex by income interaction was not significant in model 8 (Table 3), removing the sex by cognitive function interaction increased the sex by income interaction effect (ĉ=0.97; 95% CI, 0.94–1.00; *P*=0.029; Table S3), suggesting that the 2 interactions are accounting for the same outcomes. For additional sensitivity analysis, we evaluated a model with a sex by adult SES interaction, finding no evidence for an overall SES interaction. We also examined whether having a spouse or other partner accounted for any of the sex and income associations (Table S3) and found no evidence for any such influence.

## Discussion

Our results show that sex and cognitive function from youth interact significantly to predict hypertension diagnosis by middle age. Women with higher cognitive function are less likely than higher cognitively functioning men to develop hypertension, as indicated by reported doctor diagnosis. The opposite is not necessarily true at the other end of the spectrum: lower functioning women appear about as likely to develop hypertension as lower functioning men.

SES from youth did not explain the effects of the interaction, and youth SES was only associated with hypertension diagnosis before the addition of adult SES variables. This is consistent with prior work^6,25^ but is nonetheless notable, given that cognitive function and youth SES were assessed at the same time and the correlation between the two is high (*r*=0.56).

Adult SES, on the contrary, did predict hypertension diagnosis, such that higher SES individuals were less likely to have hypertension. The association with adult SES attenuated the sex-independent effect of cognitive function on hypertension and made it nonsignificant, while preserving the sex by cognitive function interaction. The foundation for this can be seen in the curves presented in Figure 2: high and middle functioning males do not appear to differ. Adult SES and cognitive function are strongly correlated (*r*=0.60 for women; *r*=0.66 for men), and our findings suggest that the antihypertensive benefit gained by those with higher cognitive function from youth that spans the sexes can be accounted for by adult family income.

Individuals with higher income were less likely to develop hypertension. Income alone did not affect the sex by cognitive function interaction, but including an interaction between sex and family income ablated the sex by cognitive function interaction. This suggests that the segment of higher cognitive functioning women, who are even less likely to become hypertensive, overlaps with the segment of higher SES women, particularly women from higher income families, who also are less likely to become hypertensive.

Both men and women in this sample with higher cognitive function from youth tended to have a higher family income (*r*=0.48) in adulthood. It is difficult to causally determine whether the higher cognitive functioning segment of women benefitted directly from having higher family income. One explanation is that income mediates some or all of cognitive function’s effect on hypertension, although an unmeasured confounder(s) could still be driving these associations. For instance, evidence from molecular genetic cognitive epidemiology suggests that cognitive function and hypertension share some genetic underpinnings.^37^

There is more evidence for the importance of lifestyle factors in explaining the associations between cognitive function and physical health. The Aberdeen Children of the 1950 cohort yielded results that were similar to ours; specifically, associations between childhood cognitive function and both stroke and coronary artery events were stronger in women.^7^ However, in their analyses, the sex by cognitive function interaction effects on stroke and CAD outcomes could be accounted for by education, not income. The Aberdeen cohort began earlier than the NLSY79 and is from the United Kingdom, not the United States, so chronological and geographic cultural cohort differences might explain this discrepancy.^38–40^

Developing hypertension is known to be robustly associated with adult SES, in particular education but to lesser degrees, with income and occupation.^18^ Women in particular seem to benefit more from having higher SES in all 3 categories, and women also appear to drive the meta-analytic association between hypertension and both income and occupation.^41^ High cognitively functioning women may not have the opportunity to progress as far as men in the workplace as men and thus might be shielded from the risks of high SES occupations: Lubinski et al^42^ observed that high cognitively functioning men work more and prioritize their job over other activities and goals, although the evidence that higher occupational status is associated with hypertension is mixed.^18^

In the context of cardiovascular disease, women are less likely to be diagnosed,^43^ and lower SES adults and women are less likely to seek preventive treatments.^44^ A key ecological reason for why lower SES adults may not seek preventive treatment is that in more socially and economically deprived areas, there is a lower concentration of and reduced access to primary care services, which is linked to increased cardiovascular disease and mortality.^45,46^ Because women tend to use primary health care and preventative services more often than men,^47^ the stronger association between cognitive function and hypertension observed among women may be influenced through the mediator of access to healthcare services. Higher cognitive function men with higher income might not put money toward health services, which we have speculated women might do^47^; instead, there might even be a tendency for men to spend some disposable income on health-harming habits, such as alcohol^48^ because men are more likely than women to drink alcohol.^49^

In general, our results are consistent with previous meta-analyses that have indicated that the effects of SES on hypertension diagnosis, as well as cardiovascular disease, are stronger and more consistent in women.^18,19^ Our results suggest that both men and women with lower cognitive function are more generally at increased risk of developing hypertension. This group is more at risk for heart disease to begin with, not only because some individuals do not as readily seek treatment. On the contrary, the effect is different at the other end of the spectrum: higher functioning women are much less likely to develop hypertension than higher functioning men.

The present study is limited by a nontrivial proportion of missing data, particularly in the adult SES variables, which reduced our available analytical sample. The analytic sample we were left with was more affluent than the average across the whole sample. This might bias our results by excluding a sector of the population wherein hypertension is more prevalent (Table S4). This bias may have underestimated the associations of sex and cognitive function with hypertension diagnosis, for when all possible individuals’ hypertension trajectories are plotted, the sex and cognitive function effects appear even stronger (Figure S1).

It was a limitation of our modeling software that we could not account for these differences with probabilistic weighting. However, prior imputation analyses suggested that our results would not be biased,^6^ and our analyses were still able to make use of ≈1000 cases of hypertension per sex.

The diagnoses in the present study were self-reported, and we were not able to cross-reference these reports with any physician records. Although we took steps to treat diagnoses and diagnosis dates conservatively, self-reported diagnoses of hypertension tend to have lower validity than those drawn from medical records.^50^ Our use of diagnosis times would likely protect our analyses from some more common issues with low specificity in self-reports of hypertension.^50^

## Perspectives

Our study supports the association between cognitive function in youth and hypertension development and finds a stronger association in women. Adult income seems to play an important, potentially mediating role in the effects. These results further our understanding of the sex and sex risk factors that associate hypertension, cognitive function, and health inequalities. Future work should aim to elucidate the different contributions of cognitive function and SES on hypertension and other relevant physical health concerns that differ between the sexes. In finding clues to alleviating the population burden of hypertension, further attention could be given to what contributes to the especially low risk in women with higher cognitive ability.

## Acknowledgments

We wish to thank Costantino Iadecola and 3 anonymous reviewers for their helpful and constructive feedback.

## Sources of Funding

This work was conducted in the University of Edinburgh Centre for Cognitive Ageing and Cognitive Epidemiology, part of the cross-council Lifelong Health and Wellbeing Initiative (MR/K026992/1). Funding from the Biotechnology and Biological Sciences Research Council, Economic and Social Research Council, and Medical Research Council (MRC) is gratefully acknowledged. This work was also supported by an MRC Mental Health Data Pathfinder award (MC_PC_17209).

## Disclosures

None.

## Supplementary Material

## Novelty and Significance

### What Is New?

- Women with higher early-life cognitive function are at lower risk for developing hypertension than their male counterparts.
- The relationship is unaffected by the inclusion of youth and adult socioeconomic status variables.
- Family income is a potential mediator of the relationship.

### What Is Relevant?

- Women with higher cognitive ability could provide insights into how to protect populations from the risk of hypertension.
- Understanding root causes and mediating relationships will open up useful avenues for future treatment and intervention.

### Summary

Higher early-life cognitive function is associated with better later-life health outcomes, including hypertension. These associations persist even when accounting for socioeconomic status, although socioeconomic status-hypertension gradients are stronger in women. We found that higher functioning women were less at risk for hypertension than their male counterparts. Income differences could account for these associations and may mediate the relationship between early-life cognitive function and hypertension.
