# Supplementary material for: Hypertension Development by Midlife and the Roles of Premorbid Cognitive Function, Sex, and Their Interaction
Source: Hypertension. 2019 Feb 19;73(4):812–9. doi: 10.1161/HYPERTENSIONAHA.118.12164 (PMC6426348; doi:10.1161/HYPERTENSIONAHA.118.12164)
Supplement: Supplementary file 1 [file hyp-73-812-s001.doc]

ONLINE SUPPLEMENT

Hypertension development by midlife and the roles of pre-morbid cognitive function, sex, and their interaction

Drew M. Altschul^1,2*^, Christina Wraw^1,2^, Geoff Der^3^, Catharine R. Gale^2,4^, Ian J. Deary^1,2^

1. The University of Edinburgh, Department of Psychology, 7 George Square, Edinburgh, EH8 9JZ, UK
2. Centre for Cognitive Ageing and Cognitive Epidemiology, University of Edinburgh, EH8 9JZ, UK
3. MRC/CSO Social & Public Health Sciences Unit, 200 Renfield Street, University of Glasgow, G2 3QB, UK
4. MRC Lifecourse Epidemiology Unit, University of Southampton, Southampton General Hospital, Southampton, SO16 6YD, UK

* corresponding author – drew.altschul@ed.ac.uk

| Table S1. Test of proportional hazards assumption for base model. | | | |  |
| --- | --- | --- | --- | --- |
|  | ρ | χ^2^ | p |  |
| Sex | -0.0194 | 1.12 | 0.290 |  |
| Cognitive Function | -0.0715 | 15.06 | < 0.001 |  |
| Youth survey age | -0.2900 | 246.19 | < 0.001 |  |
| Global |  | 278.97 | < 0.001 |  |
| ρ = the correlation between transformed survival times and the scaled  Schoenfeld residuals. | | | | |

| Table S2. Descriptive statistics of explanatory, control, and outcome variables, both split by and collapsed across sex. | | | | | | | | |
| --- | --- | --- | --- | --- | --- | --- | --- | --- |
|  |  |  |  |  |  |  |  |  |
| Analytic sample | All (N = 5251) | |  | Male (N = 2572) | |  | Female (N = 2679) | |
|  | Mean | SD |  | Mean | SD |  | Mean | SD |
| AFQT (Cognitive Function) | -0.20 | 0.99 |  | -0.16 | 1.06 |  | -0.24 | 0.93 |
| Youth SES | -0.28 | 1.08 |  | -0.24 | 1.09 |  | -0.32 | 1.06 |
| Adult SES | 0.10 | 0.77 |  | 0.10 | 0.78 |  | 0.09 | 0.76 |
| Family Income | 0.12 | 0.87 |  | 0.19 | 0.88 |  | 0.04 | 0.85 |
| Education | 0.12 | 0.97 |  | 0.06 | 0.98 |  | 0.17 | 0.96 |
| Occupation Status | 0.05 | 1.00 |  | 0.04 | 0.99 |  | 0.06 | 1.01 |
| Hypertension diagnoses, % of respondents | 1917 | 36.5% |  | 977 | 38.0% |  | 940 | 35.1% |
|  |  |  |  |  |  |  |  |  |
| Individuals missing data | All (N = 7427) | |  | Male (N = 3825) | |  | Female (N = 3602) | |
|  | Mean | SD |  | Mean | SD |  | Mean | SD |
| AFQT (Cognitive Function) | -0.30 | 1.02 |  | -0.31 | 1.07 |  | -0.28 | 0.98 |
| Youth SES | -0.42 | 1.05 |  | -0.43 | 1.02 |  | -0.40 | 1.07 |
| Adult SES | -0.18 | 0.80 |  | -0.20 | 0.82 |  | -0.14 | 0.77 |
| Family Income | -0.48 | 1.10 |  | -0.46 | 1.10 |  | -0.50 | 1.10 |
| Education | -0.25 | 1.01 |  | -0.33 | 0.99 |  | -0.18 | 1.03 |
| Occupation Status | -0.20 | 0.97 |  | -0.31 | 0.91 |  | -0.09 | 1.02 |
| Hypertension diagnoses, % of respondents | 1183 | 47.7% |  | 605 | 50.5% |  | 578 | 45.1% |

| Table S3. Sensitivity survival analyses of hypertension diagnoses. | | | | | | | |  |  |  |  |  |  |  |  |
| --- | --- | --- | --- | --- | --- | --- | --- | --- | --- | --- | --- | --- | --- | --- | --- |
|  |  | Model S1 | |  |  | Model S2 | |  |  | Model S3 | |  |  | Model S4 | |
| Predictor | ĉ | S.E. | p |  | ĉ | S.E. | p |  | ĉ | S.E. | p |  | ĉ | S.E. | p |
| Sex | **0.96** | **0.013** | **0.003** |  | **0.97** | **0.013** | **0.008** |  | **0.97** | **0.014** | **0.011** |  | **0.95** | **0.014** | **0.001** |
| Cognitive Function | 0.98 | 0.010 | 0.057 |  | **0.98** | **0.010** | **0.013** |  | 0.99 | 0.012 | 0.228 |  | 0.99 | 0.010 | 0.324 |
| Youth survey age | **0.99** | **0.003** | **0.001** |  | **0.99** | **0.003** | **0.001** |  | **0.99** | **0.003** | **< 0.001** |  | **0.99** | **0.003** | **0.001** |
| Sex * Cog Function | **0.97** | **0.016** | **0.033** |  |  |  |  |  | 0.98 | 0.016 | 0.196 |  | **0.97** | **0.015** | **0.012** |
| Youth SES | 0.98 | 0.010 | 0.123 |  | 0.99 | 0.007 | 0.099 |  | 0.99 | 0.007 | 0.102 |  | 0.99 | 0.007 | 0.145 |
| Sex * Youth SES | 0.99 | 0.014 | 0.916 |  |  |  |  |  |  |  |  |  |  |  |  |
| Family income |  |  |  |  | **0.98** | **0.010** | **0.034** |  |  |  |  |  | **0.97** | **0.009** | **< 0.001** |
| Sex * Family income |  |  |  |  | **0.97** | **0.014** | **0.029** |  |  |  |  |  |  |  |  |
| Adult SES |  |  |  |  |  |  |  |  | 0.99 | 0.015 | 0.322 |  |  |  |  |
| Sex * Adult SES |  |  |  |  |  |  |  |  | 0.98 | 0.021 | 0.311 |  |  |  |  |
| Spouse or partner status |  |  |  |  |  |  |  |  |  |  |  |  | 1.00 | 0.015 | 0.791 |
| AIC | 20018.0 | |  |  | 19997.5 | |  |  | 20013.0 | |  |  |  |  |  |
| Log-likelihood | -10003.0 | |  |  | -9992.75 | |  |  | -9999.5 | |  |  |  |  |  |
| ĉ: acceleration factor, the degree to which an outcome is accelerated after the first observation. AIC: Akaike Information Criterion. Model S4 is fitted to a smaller sample (n = 4842) than the other models, so the AIC and Log-likelihood are not given because they are not comparable to those in the other models. | | | | | | | | | | | | | | | |

| Table S4. Survival models of hypertension diagnoses stratified by sex. | | | | | | | |
| --- | --- | --- | --- | --- | --- | --- | --- |
|  |  | Men |  |  |  | Women | |
| Predictor | ĉ | S.E. | p |  | ĉ | S.E. | p |
| Cognitive Function | 0.99 | 0.012 | 0.306 |  | **0.96** | **0.014** | **0.006** |
| Youth survey age | **0.99** | **0.004** | **0.006** |  | **0.99** | **0.004** | **0.028** |
| Youth SES | 0.99 | 0.010 | 0.178 |  | 0.99 | 0.010 | 0.322 |
| Adult SES | 0.98 | 0.015 | 0.295 |  | **0.96** | **0.015** | **0.015** |
| ĉ: acceleration factor, the degree to which an outcome is accelerated after the first observation. | | | | | | | |
|  |  |  |  |  |  |  |  |


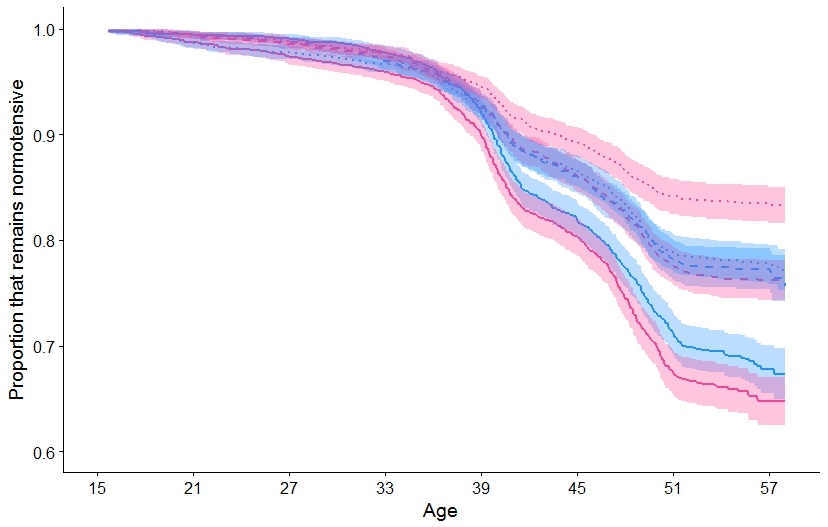


Figure S1. Kaplan-Meier curves of time to hypertension diagnosis, including all NLSY79 participants with cognitive function data. As in Figure 2, for visualisation purposes, cognitive function across all individuals was divided into tertiles. Individuals in these tertiles were subdivided by sex, producing six curves. Blue indicates men, and red indicates women. The solid line is the lowest tertile, the dashed line is the middle tertile, and the dotted line is the upper tertile. The band around each curve is the 95% confidence region.
